# Efficacy of four different hemostatic agents in thyroid surgery in reducing the amount of post-operative fluid collection

**DOI:** 10.1186/s12893-023-02172-7

**Published:** 2023-10-04

**Authors:** Thomas von Ahnen, Martin von Ahnen, Sonja Militz-Müller, Anna Süß, Josefine Schardey, Stefan Schopf, Hans-Martin Schardey, Ulrich Wirth

**Affiliations:** 1 Krankenhaus Agatharied GmbH, Hausham, Germany; 2 Ludwig-Maximilians-Universität München, München, Germany; 3 RoMed Klinik Bad Aibling, Bad Aibling, Germany

**Keywords:** Postoperative bleeding, Hemostatic agents, Thyroid surgery, Drainage volume

## Abstract

**Purposes:**

Postoperative bleeding remains a life-threatening complication in thyroid surgery. The aim was to assess the efficacy of four different hemostatic agents, Collagen-Fibrinogen-Thrombin Patch (CFTP) in two sizes (3 × 2.5 cm and 9.5 × 4.8 cm), polysaccharide particles (1 g) and Cellulose Gauze (2.5 × 5 cm) on postoperative drainage volume (DV) compared to a control group.

**Methods:**

We included from October 2007 until Mai 2011, 150 patients (30 per group) for this monocentric, retrospective case-controlled study. Patients were scheduled for a hemithyroidectomy or thyroidectomy. The primary endpoint was the postoperative DV within the first 24 h, secondary the incidence of adverse events.

**Results:**

There were no difference in demographic parameters. The mean DV (± SD) was 51.15 (± 36.86) ml in the control, 50.65 (± 42.79) ml in small (3 × 2.5 cm), 25.38 (± 23.99) ml in large CFTP (9.5 × 4.8 cm), 53.11 (± 39.48) ml in the polysaccharide particles and 48.94 (± 30.59) ml in the cellulose gauze group. DV was significantly reduced with the large CFTP (*p* < 0.05) compared to all other groups. There were no adverse events.

**Conclusions:**

We were able to demonstrate a significant reduction in the DV for the large CFTP group compared to the other collectives. Although this as being associated with not inconsiderable costs and we would only recommend its use for high-risk patients only.

## Introduction

The thyroid gland is a well perfused organ, there for a meticulous hemostasis during surgical procedures is essential [1]. To date, post-thyroidectomy hemorrhage admittedly counts as a rare but nonetheless sudden, unpredictable and potentially life-threatening event [2–5]. The incidence of postoperative bleeding after thyroid surgery ranges from 0.9% to 2.1% [3–7]. It requires immediate decompression and a subsequent surgical therapy [6]. Due to the close anatomical location of thyroid vessels, laryngeal nerves and parathyroid glands, these revision procedures are associated with an increased risk of additional complications. Morbidity particularly increases through uncontrolled and blind maneuvers from surgeons with limited experience in thyroid surgery [8].

Over the last decades numerous technical devices have been developed to improve hemostasis in thyroid surgery such as, suture ligatures, vessel ligating clips, electrocoagulation by mono- or bipolar instruments and several topical hemostatic agents (HA) [9]. HA are divided into two groups: biologically active and physically active [1]. So far, the use of HA in thyroid surgery has been widely reported in the literature [10]. But their efficacy remains controversial [10].

Studies for the biologically active agents demonstrate an advantage over standard treatment in the mean operation time, reduction of 24-h DV, time to drain removal, incidence of post-operative seroma and length of hospital stay [8, 11–16]. However, not all studies confirm these findings [17, 18]. However, physical agents don´t show advantages over conventional surgical techniques on time to drain removal, length of hospital stay and seroma formation [2, 8, 12]. Undeniably the use of HA is associated with additional costs [18].

The purpose of this monocentric retrospective case–control study is, to compare the effectiveness of four different HA at the decreasing amount of fluid collected in the thyroid cavity following thyroid resection. We compare collagen fibrinogen and thrombin patch (CFTPs) of two different sizes (3 × 2.5 cm and 9.5 × 4.8 cm), polysaccharide particles (1 g) and cellulose gauze (2.5 × 5 cm) in thyroid surgery to a control group without HA.

## Material and methods

### Study Design

This, monocentric retrospective case–control study analyzes 150 patients. All patients underwent thyroid surgery at the academic teaching hospital of Ludwig-Maximilians University in Agatharied, Hausham, Germany and were over 18 years of age. Patients with thyroid cancer, neck dissection, sternotomy, allergies, coagulopathy or drugs interfering with hemostasis were excluded from analysis. The study was approved by the local ethic committee (LMU 20–0731) and conducted according to the Declaration of Helsinki. Informed consent was obtained from all subjects.

### Surgery

All procedures were carried out by the same surgeon experienced in thyroid surgery. Patients were scheduled for hemithyroidectomy or thyroidectomy depending on the clinical presentation and sonographic findings according to the German national guidelines. For the detection of unrecognized bleeding a Valsalva maneuver was performed with pressures > 35 mmHg before wound closure. Following precise conventional hemostasis during surgery, HA have been applied. The HA have been placed into the thyroid cavity, covering at least the entrance of the recurrent laryngeal nerve (RLN) with its perineural vessels. We routinely used suction drains (10 Charr Redon, Braun, Melsungen, Germany) in every patient. Every drain was placed into one side of the thyroid cavity following resection (one drain in hemithyroidectomy and two drains in thyroidectomy). As we routinely measured the volume of the suction drains 24 h following thyroid surgery in every patient. We use this parameter as the amount of postoperative serosanguinous fluid.

### Examined collectives

**Fig. 1:**
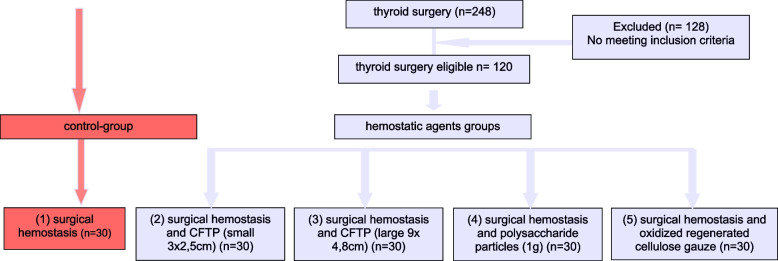
Flowchart of the trail design (CFT*P* = Collagen-Fibrinogen-Thrombin Patch)

There are five groups, each with a different hemostasis (Fig. 1). The period of medical records was from April 2008 until Mai 2011 and for the control group from October 2007 to April 2008. At that time, we received a different HA about every year from the pharmaceutical companies and our purchasing department. We continuously used those available HA on our patients for safety reasons. In addition, we chose a period in which we evermore used a suction drain, to make a statement about the effectiveness of the HA. As of 2015, our high-volume center no longer uses a suction drain for every procedure.

A sample size of 30 patients per group (total 120 patients) was estimated to generate significant results with a power > 85%. We excluded patients with thyroid cancer, neck dissection, sternotomy, allergies, coagulopathy or drugs interfering with HA to create no bias.

**Fig. 2:**
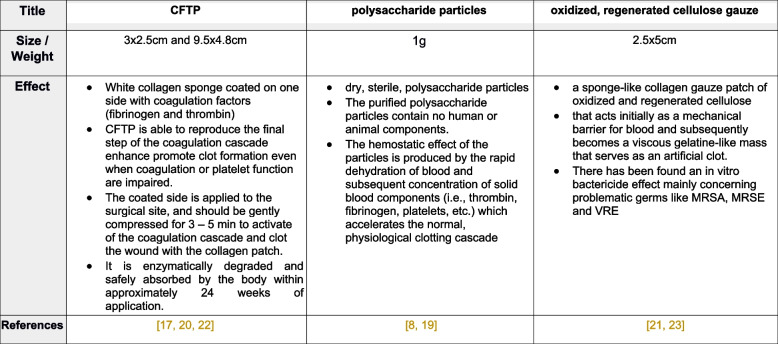
Comparison of different local hemostatic agents (CFT*P* = Collagen-Fibrinogen-Thrombin Patch, MRSA = Methicillin-resistant Staphylococcus aureus, MRSE = Methicillin-resistant Staphylococcus epidermidis, VRE = Vancomycin-resistant Enterococcus) [8, 17, 19, 20, 21, 22, 23]

Figure 2 shows the mechanism of the different HA (Fig. 2).

- *Group 1 (30 patients):* control group, with classic techniques of hemostasis (ligatures and electrocauterization)
- *Group 2 (30 patients*): small CFTP (Tachosil® 3 × 2.5 cm), CFTPs were cut into two pieces and moistened before use, pressed on the tissue with a wet gauze swab for 3 min
- *Group 3 (30 patients):* large CFTP (Tachosil® 9.5 × 4.8 cm), CFTPs were cut into two pieces and moistened before use, pressed on the tissue with a wet gauze swab for 3 min
- *Group 4 (30 patients*): polysaccharide particles (PerClot® 1 g), polysaccharide particles were used off the shelf without any additional preparation
- *Group 5 (30 patients*): oxidized, regenerated cellulose gauze (Tabotamp Fibrillar® 2.5 × 5 cm), Cellulose gauze was only divided into fine layers to increase its surface area

### Outcome parameters

The primary outcome is the DV 24 h postoperatively. Secondary outcome measures are adverse events concerning the use of HA, such as foreign body reaction, wound infection, edema or nerve entrapment. Additionally, the incidence of hypocalcemia, hypoparathyroidism, RLN palsy, difficulty swallowing, rebleeding and formation of postoperative seromas were recorded.

### Statistical analysis

Analysis of variance with post hoc Bonferroni tests is used to analyze whether there is a statistically significant difference in drain delivery volumes using different HA compared to the control group. Mean DV and standard deviation (SD) as well as range (min–max) are calculated for each group. *P* values of α ≤ 0.05 are considered statistically significant and are marked by an asterisk. Analyses were performed with SPSS 22.

## Results

### Baseline characteristics

A total of 150 patients are included, with 30 participants per group. The mean age is 59.33 years (range 22–84 years), and 70.67% (106) of participants are female. The five groups are homogeneous for age, sex, ASA score, mean duration of surgery and numbers of lobectomies and thyroidectomies (*p* > 0.05). The mean operative time is 145.8 ± 46 min, without any significant difference between the five groups (*p* = 0.52). There is no significant difference in the mean length of hospital stay (2.4 ± 0.8 days, *p* = 0.77). The mean resection volume is 74.0 ± 76.5 mg (*p* = 0.53) (Table 1.)

**Table 1:** Baseline characteristics

**Variables**	Total (*n* = 150)	surgical hemostasis (*n* = 30)	surgical hemostasis and CFTP (3 × 2.5 cm) (*n* = 30)	surgical hemostasis and CFTP (9.5 × 4.8 cm) (*n* = 30)	surgical hemostasis and polysaccharide particles (1 g) (*n* = 30)	surgical hemostasis and oxidized regenerated cellulose gauze (*n* = 30)	*P* Value
**Demographics**							
Age, y, mean ± SD	59.3 ± 11.8	57.4 ± 10.7	59.6 ± 12.7	59.8 ± 12.3	62.7 ± 11.2	57.2 ± 11.9	.29
Gender male/female, No	44/106	9/21	9/21	8/22	5/25	13/17	.16
ASA-Score, No							.85
ASA 1	71	14	15	12	13	17	
ASA 2	67	11	14	16	15	11	
ASA 3	12	5	1	2	2	2	
Lobectomy/ Thyroidectomy, No	38/112	8/22	8/22	7/23	7/23	8/22	1.0
Mean operative time, (min) ± SD	145.8 ± 46	141.7 ± 43.5	151.1 ± 47.8	137.6 ± 36.1	141.3 ± 49.8	157.5 ± 51.2	.52
Mean duration of hospital stay (d) ± SD	2.4 ± 0.8	2.5 ± 0.7	2.5 ± 0.9	2.4 ± 1.2	2.4 ± 0.7	2.3 ± 0.4	.77
Resection volume (mg) ± SD	74.0 ± 76.5	80.3 ± 78,4	63.5 ± 53.9	84.2 ± 116.1	57.2 ± 35.5	83.4 ± 75.6	.53

*ASA* American Society of Anesthesiologists, *CFTP* Collagen-Fibrinogen-Thrombin Patch

### Primary endpoint

**Fig. 3:**
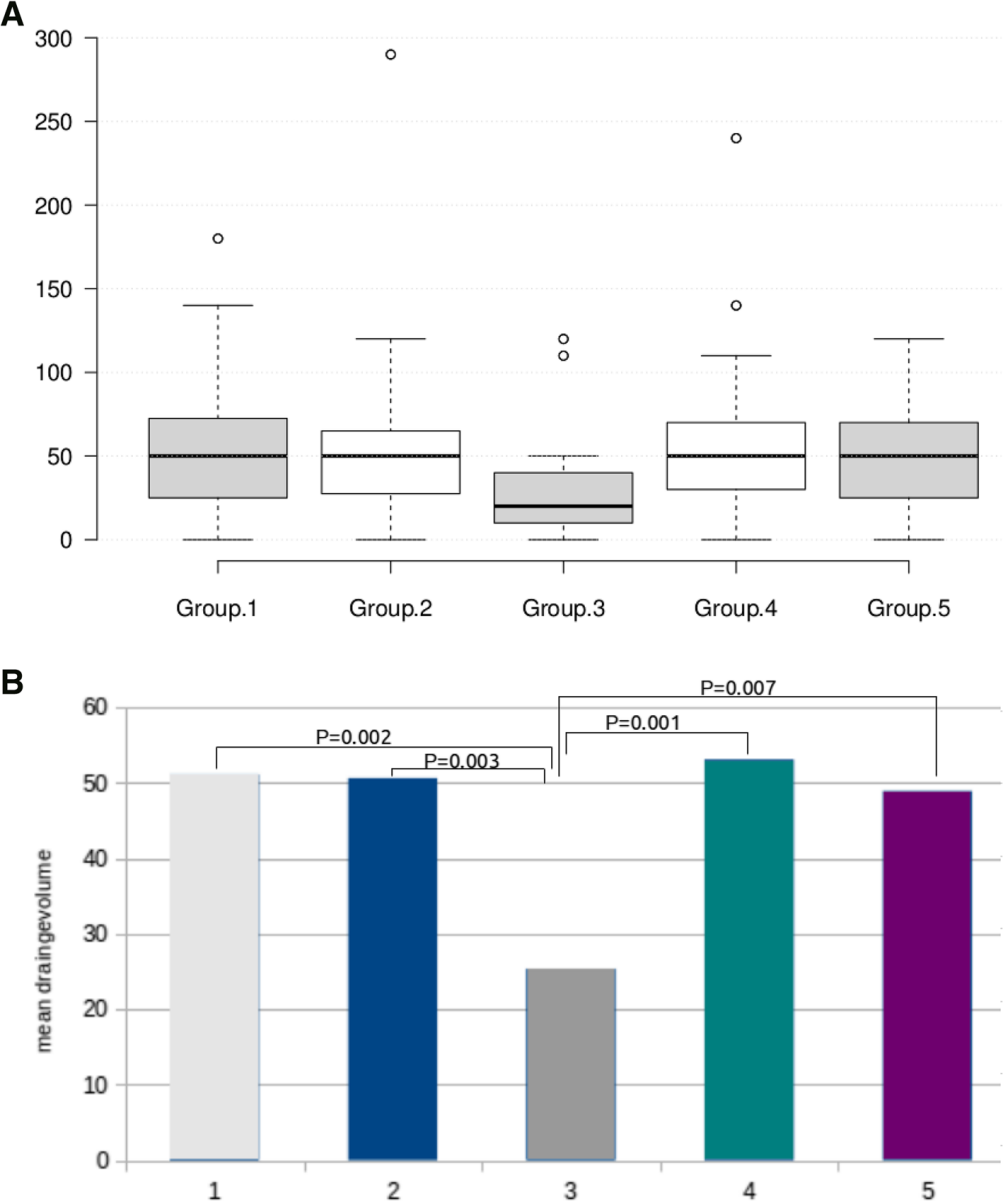
**A** Mean values and SD in drain volumes 24 h. after surgery for all groups, group 1 surgical hemostasis, group 2 surgical hemostasis and CFTP (CFT*P* = Collagen-Fibrinogen-Thrombin Patch) (3 × 2.5 cm), group 3 surgical hemostasis and CFTP (9.5 × 4.8 cm), group 4 surgical hemostasis and polysaccharide particles (1 g), group 5 surgical hemostasis and oxidized regenerated cellulose gauze. **B** Mean drainage volume 24 h after surgery, All groups differ significantly from group 3 CFTP (9.5 × 4.8 cm)* (CFT*P* = Collagen-Fibrinogen-Thrombin Patch)

The DV 24 h postoperatively is significantly lower in the large CFTP group (25.38 ml ± 23.99) compared to those in the other four groups (*p* < 0.05*). These four groups (control group 51.15 ml ± 36.86; small CFTP 50.65 ml ± 42.79; polysaccharide particles 53.11 ml ± 39.48 and oxidized, regenerated cellulose gauze 48.94 ml ± 30.59) do not differ significantly in DV (*p* > 0.05) (Fig. 3).

### Secondary endpoint

We did not observe any adverse events concerning the use of HA, such as foreign body reaction, wound infection, edema or nerve entrapment. There was no case of persistent hypoparathyroidism, difficulty swallowing, rebleeding or persistent RLN palsy. The incidence of postoperative transient hypoparathyroidism, seroma, and transient RLN palsy do not differ significantly among the groups (*p* > 0.05) (Table 2).

**Table 2:** Comparisons of primary and secondary end points

**Variables**	Total (*n* = 150)	surgical hemostasis (*n* = 30)	surgical hemostasis and CFTP (3 × 2.5 cm) (*n* = 30)	surgical hemostasis and CFTP (9.5 × 4.8 cm) (*n* = 30)	surgical hemostasis and polysaccharide particles (1 g) (*n* = 30)	surgical hemostasis and oxidized regenerated cellulose gauze (*n* = 30)	*P* Value
**Primary end point**							
Drainage amount, mL, mean ± SD		51.15 ± 36.86	50.65 ± 42.79	25.38 ± 23.99	53.11 ± 39.48	48.94 ± 30.59	< .05*
**Secondary end points**							
*Complications, No*							.60
postoperative hematoma	2	0	1	0	1	0	
seroma	1	0	1	0	0	0	
hypocalcemia	14	4	2	1	1	6	
RLN-palsy	2	1	1	0	0	0	
*Adverse events, No*							1.0
Allergic reactions	0	0	0	0	0	0	
Wound healing disorders	0	0	0	0	0	0	
Damage to the RLN	0	0	0	0	0	0	

*RLN* Recurrent laryngeal nerve, *CFTP* Collagen-Fibrinogen-Thrombin Patch

## Discussion

It is well known that postoperative hemorrhage is a rare but potentially devastating complication of thyroid surgery [3, 7] and occurs in 80% of patients within 6–23 h after surgery [4, 24]. Developed for effective intraoperative control of bleeding, various topical HA have been designed and used effectively for hemostasis in thyroid surgery [2, 8, 17, 25, 26]. But their effectiveness in preventing postoperative bleeding and in prevention of seromas remains controversial [2, 10, 27]. Auvinen et al. first reported in 1987 the effect of tranexamic acid on perioperative bleeding [28]. Later on, topical HA have been developed.

We are able to show that large CFTP can significantly reduce the 24 h DV (*p* < 0.05). Compared to the physical HA (polysaccharide particles and oxidized, regenerated cellulose gauze), CTFP is a biologically active. “CFTP is a sealant patch of equine collagen medicated with thrombin and fibrinogen. When it is in contact with blood, the fibrinogen and the thrombin are activated, giving rise to the final stage of the coagulation cascade, forming a fibrin network. The sponge firmly adheres to the tissue, and it will be absorbed completely within 12 weeks” [9].

This effect is confirmed by Tartaglia et al. This study shows a reduction of DV by CFTP of 20 ml in 24 h [9]. Erdas et al. used CFTP on the prevention of postoperative bleeding in patients with antithrombotic therapy undergoing thyroid surgery [17]. However, CFTP is not more effective than standard hemostasis in terms of postoperative bleeding, time to drain removal and length of hospital stay [18]. Due to the size of the large CFTP, a tamponade effect is also conceivable. However, we consider this to be very unlikely, since the effect is achieved via the coagulation cascade described above. To investigate whether the use of different hemostats might be able to prevent rebleeding in thyroid surgery, much larger studies or database analysis must be carried out. Scaroni et al. suspects that the use of CFTP can reduce the risk of postoperative bleeding; however, the effect in their collective of 279 patients was not significant [1].

However, some disadvantages of HA are described by Dolcetti et al. [19]. They were able to document on 84 patients a longer resorption time for HA than the data provided by the producers. They concluded, that a delay in reabsorption of the hemostatic material can cause diagnostic mistakes and affect the patient’s therapeutic path [29].

Our study has some limitations, the sample size per group is relatively small, otherwise we were able to compare four different HA to a control group. A larger sample size might have resulted in more statistically significant results, but the differences between the groups except for the large CFTP are quite small and without clinical significance in our opinion. To analyze the effect of these HA on post-thyroidectomy hemorrhage much larger cohorts with sample size > 1000 patients would be necessary. Also, the case control and retrospective character of our study can lead to possible bias and confounding factors.

The resorption time of HA was also not considered in our study. Patients with anticoagulant and antiaggregant drugs were excluded, in order to create no bias. Actually, in the clinical setting this is one of the main indications to use topical hemostatic, and should be further investigated in clinical studies. In our study, we did not find any significant difference in the resection volume, but we can imagine this as a bias factor in the case of very large resection volumes. We are also aware that according to the American guidelines there is no general recommendation for the use of drains, but within the scope of this study an objective measuring unit was required.

We also see that the data used are old. However, we still consider the data to be of interest, since bleeding after thyroid surgery is an unpredictable and life-threatening event.

We carry out a cost–benefit analysis for the use of a hemostatic agent in comparison to further surgical procedures caused by bleeding complications. CFTP was the most expensive hemostat in our study (large Tachosil® 9.5 × 4.8 cm: 439,65 €, small Tachosil® 2.5 × 3 cm: 123,57 €, Tabotamp Fibrillar® 2.5 × 5 cm: 94,77€, Perclot 1 g: 100,76€). The case value for thyroid surgery in Germany is currently 3739,35€, the relative weight for a hemithyroidectomy is 0.846 (earnings 3163,49€) and for a thyroidectomy 1.029 (earnings 3847.79€). In our opinion, the additional costs for HA and even the expensive CFTP are justified for patients at high risk for seromas or bleeding complications [5]. In case of bleeding complications and especially post thyroidectomy hemorrhage, the additional effort does not result in any additional proceeds. However, these additional costs are relevant in clinical routine. On the over hand Kallinowski et al. evaluated the benefit of fleece-bound sealing with CFTP in an open observational study. In terms of avoiding hospital resource consumption, the cost analysis showed a distinct potential for savings in favor of CFTP application. The greatest contributing factor was the savings in time spent in the operating room [30].

## Conclusion

We were able to demonstrate a significant reduction in the DV for the large CFTP group compared to the other collectives. Although this as being associated with not inconsiderable costs and we would only recommend its use for high-risk patients only.

## Data Availability

The datasets used and/or analysed during the current study available from the corresponding author on reasonable request.
